# Impact of cluster of differentiation 20 expression and rituximab therapy in classical Hodgkin lymphoma: Real world experience

**DOI:** 10.1016/j.lrr.2021.100240

**Published:** 2021-04-08

**Authors:** Khadega A. Abuelgasim, Raed Al Shammari, Saeed Alshieban, Bader Alahmari, Mohsen Alzahrani, Ayman Alhejazi, Ahmed Alaskar, Moussab Damlaj

**Affiliations:** a King Abdullah International Medical Research Center, Riyadh, Saudi Arabia; b Oncology Department, King Abdulaziz Medical City, Riyadh National Guard Health Affairs, Riyadh 11426, Saudi Arabia; c King Saud bin Abdulaziz University for Health Sciences, Riyadh, Saudi Arabia; d Internal Medicine Department, King Abdulaziz Medical City, Riyadh, Saudi Arabia; e Pathology Department, King Abdulaziz Medical City, Riyadh, Saudi Arabia

**Keywords:** Classical Hodgkin lymphoma, Cluster of differentiation 20, Rituximab, Interim positron tomography

## Abstract

The prognostic impact of CD20 expression and rituximab therapy in classical Hodgkin lymphoma (cHL) is unclear. Among 310 patients, CD20 was expressed in 66 (22%) cases. The 3-year PFS was 75.1% for CD20^+^and 70% for CD20^−^ (*p* = 0.36). The 3-year PFS was 84.7% for the rituximab group and 67.8% for the no rituximab group (*p* = 0.23). Only constitutional symptoms and positive interim PET/CT were significantly associated with worse outcome, HR 3.2 (1.14–9.01; *p* = 0.028) and 4.3 (2.27–8.1; *p* < 0.0001), respectively. Neither CD20 expression nor rituximab use significantly impacted outcome.

## 1 Introduction

Classical Hodgkin lymphoma (cHL) is a B-cell lymphoma with Reed Sternberg (RS) tumor cells expressing clonal immunoglobulin heavy and light chain gene rearrangement. Despite originating from B cells, RS cells only express cluster of differentiation 20 (CD20) in 11–35% of newly diagnosed cHL cases [1], [2], [3]. CD20 functionally couples with the B cell antigen receptor (BCR) on the surface of B lymphocytes; the BCR later internalize after the disassociation of both antigens and due to the brittle nature of CD20 epitopes, the antigen might disappear during the cellular fixation process leading to false weak or no expression by immunohistochemical (IHC) stains [4, 5]. The prognostic significance of CD20 expression in cHL is controversial. Some studies demonstrated that CD20 expression by RS cells has no significant effect on outcome [2, [6], [7], [8]]. Other authors have associated CD20 expression with worse prognosis [9], while one group suggested that CD20 expression carry a good prognosis [1].

Anti CD20 monoclonal antibodies are not considered part of the standard frontline therapy in cHL [10]. B cells, other than RS cells play a role in the pathogenesis of the tumor microenvironment in cHL, possibly via a tumor-promoting function which may justify the use of anti CD20 monoclonal antibodies in the treatment of cHL. Two phase II clinical studies tested the addition of rituximab to the backbone of adriamycin, bleomycin, vinblastine, dacarbazine (ABVD) in newly diagnosed cHL patients; in both studies rituximab was well tolerated with no additional toxicity [11, 12]. Younes at al., used R-ABVD in a total of 78 advanced stage cHL patients, 14 (18%) of whom were CD20^+^. There was a trend towards improved event free survival (EFS) in CD20^+^compared to CD20^−^ (93% vs. 77%, respectively); however, the difference was not statistically significant [11]. Kasamon et al., incorporated rituximab to ABVD in 49 cHL patients, 8% of whom were CD20^+^. The study did not observe an outcome difference of CD20^+^ vs. CD20^−^cases [12].

Strati et al., examined in a multicenter, open label, randomized phase 2 study in which R-ABVD was directly compared to ABVD alone in a total of 56 patients with advanced stage cHL [13]. They reported an improvement of 3-year EFS with no difference in overall survival (OS) with adding rituximab to ABVD in patients with CD20^+^ RS cells. The addition of rituximab to bleomycin, etoposide, doxorubicin, cyclophosphamide, vincristine, procarbazine and prednisone (R-BEACOPP) irrespective of CD20 status in patients with interim positive Positron Emission Tomography – Computed Tomography (PET/CT) in an attempt to escalate their therapy; however, failed to show improvement in outcome [14]. Patients with cHL diagnosed in the Middle East frequently present at advanced stage [15]. The aim from this analysis is twofold; first, we wished to examine the prognostic significance of CD20 expression in a cohort of 310 previously untreated cHL patients from the Middle East and North Africa region (MENA). Second, to further study whether the incorporation of rituximab specifically in CD20^+^ patients confers an improvement in progression free survival (PFS).

## 2 Materials and methods

### 2.1 Patient selection

After the due approval, we retrospectively identified all patients ≥ 14 years of age newly diagnosed with cHL at our institution in the period of 2006 – 2019. Staging was based on the Lugano classification [16]. Risk stratification was decided based on the National Comprehensive Cancer Network (NCCN) for early stage and International Prognostic Score (IPS) risk stratification for advanced stage [17, 18]. All variables including patient, disease and treatment related factors were retrieved retrospectively using the institutional electronic medical records system.

### 2.2 Histology and immunohistochemistry staining

The pre-prepared pathology slides for the identified cases were reviewed by a lymphoma Hemato-Pathologist to ensure the accuracy of the diagnosis. The reviewed material includes hematoxylin and eosin (H&E) stained slides and IHC stained slides. Cases were diagnosed as per the revised fourth edition of the World Health Organization (WHO) classification 2016 [19]. Cases of cHL diagnosed on needle core biopsy and cannot be subtyped due to small biopsy sized are labelled as classical Hodgkin Lymphoma - non otherwise specified (cHL-NOS). The IHC panel included; CD20, PAX-5, CD3, CD30, CD15, EMA, ALK, CD68 and CD163. Additional IHC stains for cases lacking one or more of these stains were performed on slides made from stored formalin-fixed parrafin-embeded tissue blocks when needed and available. In addition, in-situ hybridization (ISH) staining for EBV-EBER was used when indicated. CD20 IHC stain was applied to all cases and was considered positive when ≥10% of the RS cells showed expression [2]. These stains were provided by VENTANA© Company. The IHC and ISH staining methods and protocols of the department of pathology and laboratory medicine, King Abdulaziz Medical City, Riyadh, Saudi Arabia were applied.

### 2.3 Chemotherapy and response assessment

Patients with early stage cHL received induction regimen consistent of ABVD plus involved field radiation therapy as previously reported [20, 21]. Treatment algorithm changed over time and was previously published [22]. Majority of patients were started on ABVD except 2 patients (<1%) whom received BEACOPP front line and those with major comorbidities as shown in Table 1. Prior to availability of PET/CT from 2006 to 2010, patients were escalated to BEACOPP based on results of gallium scan at the interim stage, whereas following 2010, an interim PET/CT guided strategy was applied, except in cases where patients declined escalation due to toxicity concerns. [22]. Early unfavorable patients were managed similar to the advanced stage group, as in multiple prospective studies [23]. Rituximab was administered in CD20^+^ cases at the discretion of the treating physician. Rituximab was administered on day 1 and day 15 of each ABVD cycle and on day one of each escalated BEACCOP cycle. All patients received a minimum of two cycles of chemotherapy prior to response assessment.

**Table 1:** Baseline characteristics of the entire cohort.

Characteristic	*N* = 310
**Age, median (range)**	27 (14–89)
**Male, n (%)**	171 (55)
**Ann Arbor Stage, n (%)** **Stage I** **Stage II** **Stage III** **Stage IV**	11 (4) 85 (27) 85 (27) 129 (42)
**Advanced Stage (including Early Unfavourable)**	278 (90)
**Constitutional Symptoms, n (%)**	214 (70)
**Bulky Disease, n (%)**	58 (19)
**Lymphoma Subtype, n (%)** **Nodular Sclerosing (NS)** **Mixed Cellularity (MC)** **Lymphocyte Rich (LR)** **Lymphocyte Deplete (LD)** **Classical Hodgkin Lymphoma-NOS**	185 (60) 21 (6.7) 5 (2) 1 (0.3) 98 (31)
**CD20 Positive, n (%)**	66 (22)
**IPS Score, n (%) Stage III-IV** **0–2** **≥ 3** **Unknown**	86 (40) 112 (52) 16 (8)
**IPS Score, median (range) Stage III-IV, n (%)**	3 (0–6)
**First Line Therapy, n (%)** **ABVD** **ABVD → BEACOPP** **BEACOPP → ABVD** **Other**	234 (76) 60 (19) 2 (0.6) 14 (4.4)
**Interim PET/CT, n (%)** **Positive** **Negative**	56/209 (27) 153/209 (73
**No. of Cycles at Interim, median (range)**	2 (2–4)
**Rituximab Used, n (%)**	27 (9)
**IFRT, n (%)**	74 (24)
**Follow up, median months (range)**	51.3 (1.3–213)

**Abbreviations:** IPS, international prognostic score; ABVD, doxorubicin, bleomycin, vinblastine and dacarbazine; BEACOPP, bleomycin, etoposide, doxorubicin, cyclophosphamide, vincristine, procarbazine and prednisone; PET/CT, positron emission tomography with computed tomography; IFRT, involved filed radiotherapy.

Responses were as defined per the International Harmonization Project response criteria [24]. To determine the metabolic responses, the standardized uptake value of the liver and mediastinum was noted and update classified per Deauville criteria as ≤ liver uptake or ≤ mediastinal blood pool. Patients with update ≤ liver (i.e. Deauville 3) were deemed to have complete metabolic remission (CMR) and those with higher SUV as partial metabolic response (PMR) [25].

### 2.4 Definitions and statistical methods

Overall survival (OS) was calculated from the date of diagnosis until the date of death of any cause or last documented follow-up. PFS was calculated from the time of diagnosis until death of any cause or evidence of disease progression or relapse. Statistical analyses were performed using JMP Pro-Version 11 (SAS Institute, Cary, NC, USA) software and EZR on R commander. Baseline patient, disease and treatment related variables were reported using descriptive statistics (counts, medians and percentages). Categorical and continuous variables were compared using Pearson's chi-squared and Wilcoxon / Kruskal-Wallis, respectively. Probability of OS and PFS was computed using the Kaplan-Meier method. Group comparisons were made using the log-rank test. Time to event was calculated from the date of diagnosis until the event of interest or point of last clinical encounter, in which case the event was censored. A multivariable cox regression analysis for PFS was computed incorporating variables with a p value ≤ 0.15 from the univariable model in addition to CD20 expression and rituximab use with results expressed as hazard ratio (HR) with 95% CI. All statistical tests were declared significant at α level of 0.05 or less.

## 3 Results

### 3.1 Patients’ characteristics

We identified a total of 310 cHL patients with a median age of 27 (14–89) years and 171 (55%) were males. Two hundred and seventy eight (90%) were diagnosed at advanced stage including early unfavorable, 214 (70%) had constitutional symptoms and 58 (19%) had bulky disease at presentation. Regarding the pathological subtypes, the most common was cHL-nodular sclerosis 185 (60%). Using the cutoff ≥10%, CD20 was positive in 66 (22%) of the cases. Majority of patients received ABVD 234 (76%) and out of the 66 patients with CD20 expression, 27 (41%) received rituximab with their chemotherapy. Rituximab was administered with the start of chemotherapy in all 27 patients. Detailed patients’ characteristics are listed in Table 1.

### 3.2 Patients’ characteristics and outcome based on CD20 expression

When the entire cohort was stratified based on expression of CD20, there was no significant difference in terms of age, gender, stage at presentation, presence of constitutional symptoms or bulky disease. Nodular sclerosis (NS) subtype was more common among CD20^−^ cases (*p* = 0.0001). More patients were able to achieve interim CMR status among CD20^+^ compared to CD20^−^ at 85% vs. 69%, respectively (*p* = 0.032). The baseline characteristics stratified by expression of CD20 are detailed in Table 2.

**Table 2:** Baseline Characteristics Stratified by Expression of CD20.

Characteristic	cHL Expressing CD20 (*N* = 66)	cHL Not Expressing CD20 (*N* = 237)	P value
**Age, median (range)**	33 (14–83)	26 (14–89)	0.07
**Male, n (%)**	39 (59)	130 (55)	0.54
**Ann Arbor Stage, n (%)** **Stage I** **Stage II** **Stage III** **Stage IV**	2 (3) 15 (23) 15 (23) 34 (51)	9 (4) 67 (28) 69 (29) 92 (39)	0.33
**Constitutional Symptoms, n (%)**	43 (65)	165 (70)	0.43
**Bulky Disease, n (%)**	9 (14)	48 (20)	0.2
**Lymphoma Subtype, n (%)** **Nodular Sclerosing (NS)** **Mixed Cellularity (MC)** **Lymphocyte Rich (LR)** **Lymphocyte Deplete (LD)** **Classical Hodgkin Lymphoma-NOS**	28 (42) 5 (8) 5 (8) 0 28 (42)	153 (65) 16 (7) 0 1 (<1) 67 (28)	0.0001
**IPS Score, n (%) Stage III-IV** **0–2** **≥ 3** **Unknown**	25 (43) 41 (53.5) 2 (3.5)	111 (52) 90 (42) 13 (6)	0.27
**IPS Score, median (range) Stage III-IV, n (%)**	3 (0–5)	2 (0–5)	0.23
**First Line Therapy, n (%)** **ABVD** **ABVD → BEACOPP** **BEACOPP → ABVD** **Other**	51 (77) 12 (18) 0 3 (5)	177 (75) 47 (20) 11 (5) 2 (1)	0.77
**Interim PET/CT, n (%)** **Positive** **Negative**	6 (15) 35 (85)	50 (31) 114 (69)	0.032
**No. of Cycles at Interim, median (range)**	2 (2–3)	2 (2–4)	0.1
**Rituximab Used, n (%)**	27 (41)	0	< 0.0001
**IFRT, n (%)**	15 (23)	57 (24)	0.82
**Follow up, median months (range)**	42.6 (1.3–188)	55.2 (2.2–213)	0.21

**Abbreviations:** IPS, international prognostic score; ABVD, doxorubicin, bleomycin, vinblastine and dacarbazine; BEACOPP, bleomycin, etoposide, doxorubicin, cyclophosphamide, vincristine, procarbazine and prednisone; PET/CT, positron emission tomography with computed tomography; IFRT, involved filed radiotherapy.

**Fig. 1:**
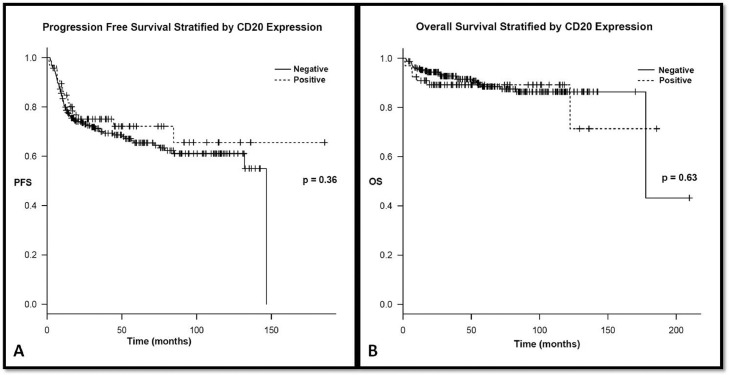
Progression and overall survival stratified based on CD20 expression.

After a median follow up of 42.6 (1.3–188) months for CD20^+^and 55.2 (2.2–213) months for the CD20^−^group, the 3-year PFS was 75.1% and 70%, respectively (*p* = 0.36). The 3-year OS was 89.2% for CD20^+^and 92.8% for CD20^−^ (*p* = 0.63) (shown in Fig. 1).

### 3.3 Impact ofrituximabtherapyin CD20^±^cHL

When the 66 CD20^+^cases were stratified based on rituximab use, there was no significant difference in terms of age, gender, stage at presentation, presence of constitutional symptoms or bulky disease. First line chemotherapy used was comparable; however more patients received involved field radiotherapy (IFRT) in the no rituximab group (*p* = 0.05). The addition of rituximab to chemotherapy did not affect the result of interim PET/CT (*p* = 0.84). The baseline characteristics of CD20^+^cases stratified by rituximab use are detailed in Table 3.

**Table 3:** Baseline Characteristics of cd20 expressing classical Hodgkin lymphoma stratified by rituximab therapy.

Characteristic	Rituximab Cohort (*N* = 27)	No Rituximab Cohort (*N* = 39)	P value
**Age, median (range)**	31 (17–69)	33 (14–83)	0.84
**Male, n (%)**	13 (48)	26 (67)	0.13
**Ann Arbor Stage, n (%)** **Stage I** **Stage II** **Stage III** **Stage IV**	1 (4) 7 (26) 4 (15) 15 (55)	1 (3) 8 (20) 11 (28) 19 (49)	0.63
**Constitutional Symptoms, n (%)**	17 (63)	26 (67)	0.76
**Bulky Disease, n (%)**	3 (11)	6 (15)	0.62
**Lymphoma Subtype, n (%)** **Nodular Sclerosing (NS)** **Mixed Cellularity (MC)** **Lymphocyte Rich (LR)** **Classical Hodgkin Lymphoma-NOS**	12 (44.5) 1 (4) 2 (7) 12 (44.5)	16 (41) 4 (10) 3 (8) 16 (41)	0.78
**IPS Score, n (%) Stage III-IV** **0–2** **≥ 3** **Unknown**	5 (26) 14 (74) 0	12 (40) 16 (53) 2 (7)	0.26
**IPS Score, median (range) Stage III-IV, n (%)**	3 (1–5)	3 (0–5)	0.59
**First Line Therapy, n (%)** **ABVD** **ABVD → BEACOPP** **Other**	21 (78) 5 (18) 1 (4)	30 (77) 7 (18) 2 (5)	0.96
**Interim PET/CT, n (%)** **Positive** **Negative**	3 (14) 19 (86)	3 (16) 16 (84)	0.84
**No. of Cycles at Interim, median (range)**	2 (2–3)	2 (2–3)	0.72
**IFRT, n (%)**	3 (11)	12 (31)	0.05
**Follow up, median months (range)**	36 (6.8–116)	55.8 (1.3–188)	0.07

**Abbreviations:** IPS, international prognostic score; ABVD, doxorubicin, bleomycin, vinblastine and dacarbazine; BEACOPP, bleomycin, etoposide, doxorubicin, cyclophosphamide, vincristine, procarbazine and prednisone; PET/CT, positron emission tomography with computed tomography; IFRT, involved filed radiotherapy.

**Fig. 2:**
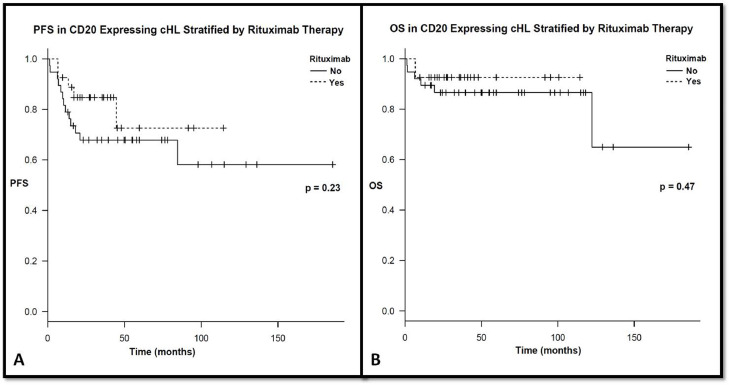
Progression and overall survival stratified based on rituximab therapy.

After a median follow up of 36 (6.8–116) months for rituximab group and 55.8 (1.3–188) months for the no rituximab group, the 3-year PFS was 84.7% and 67.8%, respectively (*p* = 0.23). The 3-year OS was 92.6% for rituximab group and 86.6% for no rituximab group (*p* = 0.47) (shown in Fig. 2).

### 3.4 Cox regression for factors influencing PFS

On univariable analysis looking at factors that may influence outcome, we found that male gender HR 1.36 (0.91–2.03; *p* = 0.13); age HR 1.01 (1.01–1.03; *p* = 0.0005); the presence of constitutional symptoms: HR 2.71 (1.58–4.64; *p* = 0.0003) and interim PET/CT HR 4.41 (2.39–8.14; *p* = < 0.0001) were associated with a p value of < 0.2 and were thus entered into the multivariable model. CD20 expression and rituximab use was also entered into the model to further verify their impact, if any. The presence of constitutional symptoms HR 3.2 (1.14–9.01; *p* = 0.028) and positive interim PET/CT HR 4.3 (2.27–8.1; *p* < 0.0001) were significantly associated with worse outcome at the multivariable stage. No significant association of CD20 expression or rituximab therapy use was observed. The univariable and multivariable analysis of different risk factors in relation to PFS are shown in Table 4.

**Table 4:** Cox regression for factors influencing progression free survival.

	Variable	Univariable HR (95% CI; P value)	Multivariable HR (95% CI; P value)
PFS	Male Gender	1.36 (0.91–2.03; 0.13)	0.8 (0.42–1.51; 0.49)
	Age (continuous) at Dx	1.01 (1.01–1.03; 0.0005)	1.01 (0.99–1.03; 0.17)
	Bulky Disease	1.02 (0.62–1.69; 0.93)	
	Constitutional Symptoms	2.71 (1.58–4.64; 0.0003)	**3.2 (1.14–9.01; 0.028)**
	High IPS (≥ 3)	1.17 (0.71–1.91; 0.53)	
	CD20 Expression	0.79 (0.47–1.31; 0.36)	0.83 (0.25–2.73; 0.76)
	Rituximab Use	0.56 (0.23–1.37; 0.21)	0.98 (0.19–4.9; 0.98)
	Interim PET/CT Positive	4.41 (2.39–8.14; < 0.0001)	**4.3 (2.27–8.1; < 0.0001)**
	IFRT Use	0.73 (0.45–1.18; 0.2)	

**Abbreviations:** Dx, diagnosis; IPS, international prognostic score; PET/CT, positron emission tomography with computed tomography; IFRT, involved filed radiotherapy.

## 4 Discussion/conclusion

Classical Hodgkin lymphoma is a highly curable malignancy and much effort over the last decade has been made to offer risk adapted therapy in order to maintain efficacy while decrease short and long term toxicity. The WHO classification of HL includes two distinct subsets; classical and nodule lymphocyte predominant (NLP) [19]. Both subtypes are derived from B-cells however with very differing histopathology, IHC pattern and even clinical outcome. NLPHL B-cells universally express CD20 whereas this is evident in only a minority of cHL patients. Furthermore, the significance of CD20 expression in cHL is less clear and its therapeutic implications are even more elusive. This is in contract to other B-driven diseases such as B-acute lymphoblastic leukemia where CD20 expression conferred an adverse prognostic factor and the use of the monoclonal antibody rituximab or ofatumumab has mitigated some of this risk [26], [27], [28].

Herein, we observed that the incidence of CD20 expression among patients from the MENA region was 22% of newly diagnosed cHL cases, which is comparable to what was reported by other groups [1, 2]. In our study, we used a cutoff value of ≥10% RS cells to define CD20 positivity. Tzankov et al. also used a cutoff ≥10% and the incidence of CD20^+^ cases was 20% whereas Rassidakis et al., used any positivity (a cutoff of >0%) of RS cells 132/598 (22%) of cases were CD20^+^. Other groups however such as Watanabe et al., reported a higher incidence of 35% among a smaller cohort of 51 patients whereas the Memorial Sloan-Kettering Cancer Center (MSKCC) group found that only 28 (11%) were CD20^+^ among a cohort of 248 patients [1, 29].

In the present study, we found no major differences in the presenting clinical features of CD20^+^ and CD20^−^ newly diagnosed cases of cHL. There were a significantly higher percentage of patients with negative interim PET/CT whom did not express CD20, 85% in vs. 69%, respectively (*p* = 0.032). Interestingly, this has not translated into a significant difference in the outcome with regards to PFS or OS the 3-year PFS was 75.1% and 70%, respectively (*p* = 0.36). The 3-year OS was 89.2% for CD20^+^and 92.8% for CD20^−^ (*p* = 0.63). This is in contrast to what was reported by Portlock et al., where CD20^+^ positive patients treated with ABVD fares substantially worse than their CD20^−^ counterparts [9].

We also reported the impact of rituximab in the front line therapy of CD20^+^ patients in the real world setting. We observed there was a non-significant trend to better outcomes in rituximab-treated patients with CD20+ cHL compared to those who did not receive rituximab. Previously, a phase II study examined the combination of rituximab with ABVD in newly diagnosed cHL and demonstrated to be safe with promising EFS at 5 years of 83% [11]. A number of observations from the Younes at al., study are worth noting. First, no comparative arm was available to further define the added role of rituximab. Second, rituximab was given to all patients irrespective of CD20 status. Lastly, rituximab administration was weekly for six weeks whereas in our cohort it was given every other week for a total of 12 doses.

In the present cohort, the presence of constitutional symptoms as well as the presence of residual disease at the interim stage detected by PET/CT conferred an adverse factor for PFS at the multivariable analysis stage. This is in line of what we reported previously from our center as well as a number of other reports on the predictive role of interim PET/CT and its potential role to offer risk adaptive therapy [22, 23, 30]. Gallamini et al., examined the role of rituximab incorporation into BEACOPP therapy as part of risk-adapted therapy in patients with positive interim PET/CT following ABVD [31]. Similarly, Brochmann et al., examined the addition of rituximab in an escalated BEACOPP backbone in patients failing to achieve CMR [32]. The rationale behind this randomization is the impressive activity demonstrated by the GHSG using single agent rituximab. In both these trials however, rituximab did not improve PFS in this high risk group of cHL patients.

This analysis carries a number of limitations, particularly with regards to its retrospective single center design and including many advanced stage patients. Rituximab use was at the discretion of the treating physician but only patients whom were CD20^+^ received rituximab. Moreover, the use of IFRT might have contributed to the difference in the outcome of CD20^+^ case who did not receive rituximab. That said, given the small number of cases, multivariable analysis was not feasible. In spite of these limitations, a number of strengths are worth highlighting. First, this was a large cohort of patients with mature follow up where almost all events are expected to take place. Second, it reflected the incidence of CD20 expression in patients from the MENA region where to our knowledge has not been reported previously. Third, we observed that CD20 expression was not associated with any baseline characteristics nor did not influence clinical outcome. Finally, we report that rituximab did not alter the outcome of CD20^+^ cHL thus it should not be used in routine clinical practice outside the setting of a clinical study. In conclusion, our retrospective real world data on CD20 expression is not associated with different PFS or OS in patients treated with equivalent regimens and that incorporation of rituximab in CD20^+^cHL has no significant impact on outcome, and should be restricted to clinical trials. The presence of constitutional symptoms and interim PET/CT positivity were associated with worse outcome.

## Author Contributions

KAA: Conceptualization, Methodology, Data acquisition, Writing-Original draft preparation

RA: Conceptualization, Data acquisition, Writing-Original draft preparation

SA: Conceptualization, Data acquisition, Writing-Reviewing and editing

BA: Writing-Reviewing and editing

MA: Writing-Reviewing and editing

AA: Supervision Writing-Reviewing and editing

AA: Supervision, Writing-Reviewing and editing

MD: Conceptualization, Supervision, Data acquisition, Formal analysis, Writing-Reviewing and editing

## Declaration of Competing Interest

The authors declare no conflict of interest.
